# A Survey of 1144 ECT Recipients, Family Members and Friends: Does ECT Work?

**DOI:** 10.1111/inm.70109

**Published:** 2025-08-10

**Authors:** John Read, Lucy Johnstone, Sarah Price Hancock, Chris Harrop, Lisa Morrison, Sue Cunliffe

**Affiliations:** ^1^ Department of Psychology and Human Development, University of East London London UK; ^2^ Clinical Psychologist and Independent Researcher Bristol UK; ^3^ Ionic Injury Foundation San Diego USA; ^4^ Clinical Psychologist and Independent Researcher Woking UK; ^5^ Lisa Morrison Training and Consultancy Belfast UK; ^6^ Independent Researcher Worcester UK

**Keywords:** efficacy, electroconulsive therapy, patient survey, shock therapy

## Abstract

The last placebo‐controlled ECT trial for depression occurred in 1985. While awaiting trials that meet today's standards of evidence‐based medicine, this paper presents the responses, to an online survey, of 858 ECT recipients and 286 family members and friends, from 44 countries, on five effectiveness measures. The majority (55%–71%) reported either no benefit or a negative outcome on the five measures. The percentages reporting some benefit were: helped the specific problem for which ECT was given—45%; improved mood—41%; generally ‘helpful’—41%; improved ‘quality of life’—29%; reduced suicidality—33%. Respondents were, unusually, given the option, on four of these measures, to report that the variable had been made worse. The results were: specific problem made worse—37%; worse mood—29%; reduced quality of life—62%; and increased suicidality—19%. The findings were consistent with responses from family and friends. It is striking that nearly half (49%) reported that their quality of life was made ‘much worse’ (22%) or ‘very much worse’ (27%) by ECT. A partial explanation of this alarming outcome is that quality of life encompasses the adverse effects of ECT alongside any benefits. In conjunction with the absence of evidence that ECT is more effective than placebo, and the known long‐term adverse effects on memory, these survey findings lead us to recommend a suspension of ECT in clinical settings pending independent large‐scale placebo‐controlled studies to determine whether ECT has any effectiveness relative to placebo, against which the many serious adverse effects can be weighed.

## 1 Introduction

Electroconvulsive therapy usually involves between 6 and 12 administrations of electricity to the brain, under general anaesthesia, over 3 or 4 weeks, in order to cause tonic–clonic seizures. A review found ‘large variation between continent, countries and regions in utilization, rates and clinical practice’ (Leiknes et al. 2012). Audits in England found 12‐fold (Read et al. 2018) and 47‐fold (Read et al. 2021) differences in the rates of ECT usage between the highest and lowest using areas.

### 1.1 Effectiveness for Depression

Such variability suggests a range of opinions among psychiatrists about this controversial intervention (Read, Cunliffe, et al. 2019). A meta‐analysis found that researchers' views varied from ‘probably ineffective but certainly causes brain damage, through to those who think it is the most effective treatment in psychiatry and completely safe’ (UK ECT Review Group 2003, 799).

Nevertheless, most ECT researchers and prescribers believe ECT is effective, especially for severe depression. The USA's Food and Drug Administration, however, requires notices to be displayed next to ECT machines stating ‘The long term safety and effectiveness of ECT has not been demonstrated’ (Food and Drug Administration 2020).

Some of the controversy about effectiveness for depression stems from the absence of robust evidence that it is more effective than placebo. A review focussed on placebo responses to ECT for depression (Rasmussen 2009), found ‘an unexpectedly high rate of response in the sham [SECT] groups’. Subsequent reviews of ECT for depression found only 11 placebo‐controlled studies comparing ECT with ‘sham’/‘simulated’ ECT (SECT), in which the general anaesthetic is administered but the electricity, and therefore the convulsion, are withheld (Read and Bentall 2010; Read et al. 2013; Read, Kirsch, and McGrath 2019). The most recent of the 11 studies was in 1985 (Gregory et al. 1985). Unsurprisingly, the reviews found that all the studies failed to comply with today's methodological standards. The 2019 review (co‐authored by Dr. Irving Kirsch, Associate Director of Harvard Medical School's Programme in Placebo Studies) concluded:

Only two studies describe their randomisation process and tested their blinding. None were genuinely double‐blind. Only four reported any ratings by patients. None assessed Quality of Life. The studies were small, involving between eight and 77 participants, with an average of 37.2. Four of the 11 found ECT significantly superior to SECT at the end of treatment, five found no difference and two found that psychiatrists reported a difference and patients did not. Neither of the only two high Quality studies reporting data at 1 or 6 months post‐treatment produced a significant difference between ECT and SECT and, when combined, they produced a very small pooled effect size (0.017) in favour of SECT.

Proponents of ECT have published vigorous critiques of these reviews, arguing that ECT is very effective for depression (Gergel et al. 2021; Meechan et al. 2021). The critiques have been countered equally strongly (Read 2022; Read et al. 2022; Read and Moncrieff 2022).

One argument made by the critiques was that although there is no placebo‐controlled evidence, other types of studies have established that ECT is effective, for instance comparisons to anti‐depressants. A review of all such non‐placebo studies between 2009 and 2016 (Read and Arnold 2017) found, however, that:

Of the 91 studies, only 2 aimed to evaluate the effectiveness of ECT. Both were severely flawed. None of the other 89 produced robust evidence that ECT is effective for depression, primarily because at least 60% maintained ECT participants on medication and 89% produced no meaningful follow‐up data beyond the end of treatment.

### 1.2 Effectiveness for Schizophrenia

There is also a lack of evidence that ECT is effective for people diagnosed with schizophrenia. A 2019 analysis of Cochrane reviews concluded that ‘What is common in all versions of these Cochrane reviews is that in spite of seven decades of clinical use of ECT for people with schizophrenia, there still is a lack of strong and adequate evidence regarding its effectiveness’ (Shokraneh et al. 2019). There have been two placebo‐controlled studies on people diagnosed with schizophrenia since 2000 (Melzer‐Ribeiro et al. 2024; Ukpong et al. 2002). Neither found any difference in effectiveness between ECT and SECT, either at the end of treatment, or at follow up.

### 1.3 Effectiveness for Suicide

It is often claimed that ECT prevents suicide. A meta‐analysis by the UK Government's ECT Review Group (2003), however, stated: ‘Although ECT is sometimes thought to be a lifesaving treatment, there is no direct evidence that ECT prevents suicide’. An investigation by the New Zealand Government found ‘no definitive randomised evidence that ECT prevents suicide’ (Ministry of Health 2004). Several studies, mostly quite recent, found that people receiving ECT are significantly *more* likely to kill themselves than people not receiving ECT (Jorgensen et al. 2020; Munk‐Olsen et al. 2007; Sharma 1999; Tsai et al. 2021). Other recent studies have found either a slight difference in favour of ECT (Kaster et al. 2022; Ronnqvist et al. 2021), or no difference (Peltzman et al. 2020; Watts et al. 2022).

### 1.4 Asking Patients

The relatively few studies that have asked patients to rate outcomes themselves have tended to focus on adverse effects rather than positive ones (Guruvaiah et al. 2017; Johnstone 1999; Rose et al. 2003). Three of the four 11 pre‐1986 placebo‐controlled studies that produced greater depression reduction in the ECT group on ratings by psychiatrists, asked patients to rate effectiveness themselves. Two studies found no difference between ECT and placebo (Freeman et al. 1978; Johnstone et al. 1980). The third failed to report the patients' ratings that they had collected (Brandon et al. 1984).

A 2009 Patient Questionnaire by the UK's Royal College of Psychiatrists (RCPsych) included no effectiveness questions. Nevertheless, 20% of patients thought the issue was important enough to spontaneously offer opinions. Most (72%) were interpreted, by psychiatrists, as indicating that ECT ‘had helped them’. Six (8%) of the patients wrote that they would not want to receive ECT again, including one who added: ‘I have never met a patient who has benefited from the experience’ (Rayner et al. 2009). A more recent RCPsych survey (RCPsych 2016) did include a question, ‘Did ECT help you?’, and reported that 77% answered ‘yes’, 12% selected ‘no’ and 11% did not know or could not remember. The option of ECT making the problem worse was not offered. Forced yes/no responses to leading statements tend to produce positive reports of services and treatments (Dunsch et al. 2018).

When 49 ECT recipients in Dublin were asked ‘Was ECT helpful?’, 23 (47%) responded ‘very helpful’ and 8 (16%) selected ‘not at all’ (Rush et al. 2007). Again, there was no option for reporting that ECT had made things worse. A meta‐synthesis of 16 qualitative studies of patients’ perspectives (Wells et al. 2020) found a range of experiences, including long‐term benefits, short‐lived improvement, no positive effects and damage to memory and other cognitive functions.

There has been even less research into the views of family members about effectiveness, and none on the views of friends. A 2019 review found ‘very little research conducted in terms of carer perspectives of ECT’ (Griffiths and O'Neill‐Kerr 2019). One study, of 27 relatives in England interviewed by the psychiatrists who had administered the ECT, reported only that ‘The majority expressed the view that ECT was beneficial’ (Guruvaiah et al. 2017).

## 2 Methods

This project employed the same methodology as online surveys about other psychiatric treatments (Cartwright et al. 2016; Larsen‐Barr et al. 2018, Moncrieff et al. 2024; Read et al. 2014, 2016, 2017; Read, Morrison, et al. 2023; Read and Sacia 2020; Read and Williams 2019). The project was approved by the Ethics and Integrity Sub‐Committee of the University of East London.

### 2.1 Instrument

A new questionnaire, ‘Survey of people who have had Electroconvulsive Therapy, and their family and friends’ [SECTAFF], includes questions based on ECT research and the experiences of the three members of the research team who have had ECT. *Mind*, the UK's largest mental health charity, commented on a draft. There are quantitative questions, with yes/no/don't know, multiple choice, or Likert scale responses, and qualitative questions inviting written responses.

The questionnaire asks about the year, country, type and number of ECTs; reasons ECT was given; positive and adverse effects; monitoring of adverse effects; and information given before ECT. Participants had to be over 18 and either have had ECT (not in the past 4 weeks) or be ‘a friend or relative with an understanding of the impact of ECT’ on the person concerned. In order to avoid ECT recipients feeling pressured to participate, and prevent possible bias from the involvement of ECT staff, the Introduction states ‘Mental health professionals must neither invite their patients/clients to complete the survey nor complete it on behalf of their patients/clients’.

A Participant Information Sheet at the outset of the survey included sources of support in case participants are distressed by the content.

#### 2.1.1 Effectiveness Questions

The questions reported on in this paper were the five questions about effectiveness:

- ‘How would you describe the problems/symptoms for which ECT was prescribed, just after the end of the treatments, compared to just before?’—on a 7‐point Likert scale from ‘very much better’ to ‘very much worse’;
- ‘Overall, how helpful was ECT?’—on a 4‐point scale from ‘Not at all’ to ‘Very’;
- ‘How did ECT affect your overall quality of life?’—on a 7‐point scale from ‘very much improved’ to ‘very much worse’;
- ‘During the treatment, what effect did ECT have on your mood?’—with a 5‐point scale from ‘much better’ to ‘much worse’;
- ‘During the treatment, what effect did ECT have on how suicidal you felt?’—with three responses, ‘less suicidal’, ‘no difference’ or ‘more suicidal’.

### 2.2 Procedure

The questionnaire was disseminated, via the online survey tool Qualtrics, from January to September 2024. The researchers contacted mental health organisations in all five continents. For example, the 44 national groups listed as members of Mental Health Europe (www.mentalhealtheurope.org) received an emailed announcement with a request to disseminate the survey to their members and other mental health groups in their countries. The survey was also disseminated on social media. No translations were provided.

### 2.3 Data Analysis

1211 surveys were returned with at least some questions answered. 55 of 63 repeat responses (identified by IP address) were deleted because the demographics and/or responses were very similar. Twelve responses were deleted because of grossly discrepant responses (e.g., last ECT at age 16, first at 100 years), 'straight‐lining' on at least three questions (selecting the same option for a list of multiple items, e.g., ‘severe’ for all 27 side effects), being a recipient's nurse (see Section 2.1), or because it was obvious that more than one relative of the same patient had responded.

Removing these 67 respondents left 1144 for analysis. Most (837, 73.2%) completed the entire survey; the other 307 (26.8%) left one or more questions unanswered.

The quantitative questions were analysed in relation to electrode placement (bilateral vs. unilateral), using chi squared tests (*X* ^2^). These analyses were also conducted on just those recipients who had received only one ECT course (thereby omitting patients who may have had different electrode placements in different courses). The numbers of reports from family/friends who had had only one course (15–24) were too small for analysis.

## 3 Results

### 3.1 Sample Characteristics

#### 3.1.1 Demographics

The 1144 respondents comprised 858 ECT recipients and 286 family members (216) or friends (70). The most common types of relative were daughter (19.4%) and mother (14.8%) of an ECT recipient. Respondents were from 44 countries. Table 1 lists the 16 countries providing at least 1% of the total. Countries providing between one and five respondents were: Austria, Belgium, Brazil, Bulgaria, Chad, Colombia, Czech Republic, Egypt, El Salvador, Greece, Guatemala, Hungary, Iceland, Iran, Iraq, Israel, Italy, Lithuania, Mexico, Pakistan, Poland, Saudi Arabia, Slovakia, South Korea, Turkey, Turkmenistan, Uruguay, Venezuela.

**TABLE 1:** Country where last ECT occurred.

Country	ECT recipients (858)	Recipients reported by family/friends (286)	Total (1144)
USA	399 (46%)	107 (37%)	506 (44%)
UK	122 (14%)	81 (28%)	203 (18%)
Australia	92 (11%)	17 (6%)	109 (10%)
Canada	65 (8%)	11 (4%)	76 (7%)
Spain	18 (2%)	14 (5%)	32 (3%)
New Zealand	20 (2%)	5 (2%)	25 (2%)
Ireland	14 (1.6%)	6 (2%)	20 (2%)
Denmark	10 (1%)	5 (2%)	15 (1%)
Netherlands	9 (1%)	3 (1%)	12 (1%)
Norway	11 (1%)	1 (0%)	12 (1%)
Sweden	10 (1%)	2 (1%)	12 (1%)
Germany	6 (1%)	3 (1%)	9 (1%)
Finland	9 (1%)	0	9 (1%)
India	8 (1%)	0	8 (1%)
France	4 (0%)	3 (1%)	7 (1%)
S. Africa	5 (1%)	2 (1%)	7 (1%)

The majority of respondents were white, and most were female (see Table 2). The average age at time of last ECT was 41.9 (recipients) and 41.7 (family/friends), ranging from 12 to 87. Table 3 shows that most had their first and last course between 2010 and 2024.

**TABLE 2:** Sex, ethnicity and age.

	ECT recipients^a^	Recipients reported by family/friends^b^
Sex		
Female	602 (73%)	181 (68%)
Male	189 (23%)	80 (30%)
Non‐binary	35 (4%)	6 (2%)
Ethnicity		
White	727 (87%)	243 (89%)
Multiple/Mixed	30 (4%)	6 (2%)
Hispanic	19 (2%)	12 (4%)
Asian	27 (3%)	3 (1%)
‘Other’	21 (3%)	6 (2%)
Black/African/Caribbean	7 (1%)	2 (1%)
Age at time of last ECT	Mean 37.4 (SD 13.2) Range 12–79	Mean 41.7 (SD 16.3) Range 15–87

^a^ Between 814 and 831 answered these questions.

^b^ Between 242 and 272 answered these questions.

**TABLE 3:** Year of first and last ECTs.

	Recipients		Reported by family/friends	
	First ECT (822)	Last ECT (786)	First ECT (256)	Last ECT (152)
2020–2024	209 (25.4%)	282 (35.9%)	64 (25.0%)	55 (36.2%)
2010–2019	324 (39.4%)	292 (37.1%)	81 (31.6%)	34 (22.4%)
1999–2009	211 (25.7%)	160 (20.4%)	49 (19.1%)	30 (19.7%)
1970–1989	58 (7.1%)	39 (5.0%)	34 (13.3%)	18 (11.8%)
1950–1969	20 (2.4%)	13 (1.7%)	28 (10.9%)	15 (9.9%)

#### 3.1.2 Number and Type of ECTs

Of the 1025 responding to the relevant question, 317 (30.9%) had had only one course of treatments, 314 had had between 2 and 5 (30.6%), 138 between 6 and 10 (13.5%) and 256 (25.0%) ‘more than 10’. Of the 964 people responding to the question about number of individual treatments in most recent session, 160 (16.6%) had received between 1 and 5, 339 (35.2%) had received 6–10, 302 (31.3%) between 11 and 20, and 163 (16.9%) ‘more than 20’. Of the 653 people who knew which electrode placement was used, 146 reported unilateral (22.4%) and 507 bilateral (77.6%).

#### 3.1.3 Reasons for ECT

When asked to select one or more reasons why ECT had been given, 74.3% chose ‘Depression’, 17.2% ‘Psychosis/schizophrenia’, 15.3% ‘Bipolar disorder/mania’, 7.8% ‘Catatonia’, 12.8% ‘Other’ and 5.7% ‘Don't know’.

### 3.2 How Would You Describe the Problems/Symptoms for Which ECT Was Prescribed, Just After the End of the Treatments, Compared to Just Before?

Table 4 shows that for 44.5% of ECT recipients, and 45.1% of the people reported on by family/friends, the problem for which ECT had been given improved to some extent (‘minimally’, ‘much’ or ‘very much’). However, 36.6% of recipients and 42.4% of family/friends thought the problem had been made worse. Nearly one in five (19.4%) of both groups selected ‘very much worse’; whereas ‘very much improved’ was selected by 10.0% of recipients and 14.9% of family/friends.

**TABLE 4:** How would you describe the problems/symptoms for which ECT was prescribed, just after the end of the treatments, compared to just before?

	Recipients (*n* = 808)	Family/Friends (*n* = 248)
1. Very much worse	157 (19.4%)	48 (19.4%)
2. Much worse	89 (11.0%)	36 (14.5%)
3. Minimally worse	50 (6.2%)	21 (8.5%)
4. No change	152 (18.8%)	31 (12.5%)
5. Minimally improved	156 (19.3%)	41 (16.5%)
6. Much improved	123 (15.2%)	34 (13.7%)
7. Very much improved	81 (10.0%)	37 (14.9%)
Mean (SD)	3.93 (1.99)	3.93 (2.12)

### 3.3 Overall, How Helpful Was ECT?

About one in five of both recipients (18.2%) and family/friends (21.0%) thought the ECT had been ‘very helpful’ (see Table 5). Most recipients (58.5%) and family/friends (60.1%) described ECT as ‘not at all helpful’.

**TABLE 5:** Overall, how helpful was ECT?

	Recipients (*n* = 802)	Family/Friends (*n* = 248)
1. Very helpful	143 (17.8%)	52 (21.0%)
2. Somewhat helpful	93 (11.6%)	26 (10.5%)
3. Slightly helpful	97 (12.1%)	21 (8.5%)
4. Not at all helpful	469 (58.5%)	149 (60.1%)

### 3.4 How Did ECT Affect Your Overall Quality of Life?

Table 6 shows that for 29.1% of ECT recipients, and 32.3% of people reported on by family/friends, ECT improved ‘overall quality of life’ (‘minimally’, ‘much’ or ‘very much’). Most recipients (62.3%), and most family/friends (61.0%), reported that ECT made overall quality of life worse. Approximately one in four recipients (27.5%) and about one in three family/friends (31.5%) reported that ECT made quality of life ‘very much worse’.

**TABLE 6:** How did ECT affect your overall quality of life?

	Recipients (*n* = 793)	Family/Friends (*n* = 251)
1. Very much improved	66 (8.3%)	36 (14.3%)
2. Much improved	95 (12.0%)	22 (8.8%)
3. Minimally improved	70 (8.8%)	23 (9.2%)
4. No change	68 (8.6%)	17 (6.8%)
5. Minimally worse	105 (13.2%)	26 (10.4%)
6. Much worse	171 (21.6%)	48 (19.1%)
7. Very much worse	218 (27.5%)	79 (31.5%)
Mean (SD)	4.81 (2.13)	4.73 (2.22)

### 3.5 During the Treatment, What Effect Did ECT Have on Your Mood?

Table 7 shows that 41.0% of ECT recipients, and 41.9% of family/friends reported that mood was better (‘somewhat’ or ‘much’) because of ECT. However, 29.4% of recipients and 33.9% of family/friends reported either ‘Somewhat worse’ or ‘Much worse’. For both groups the modal response was ‘About the same’.

**TABLE 7:** During the treatment, what effect did ECT have on your mood?

	Recipients (*n* = 800)	Family/Friends
1. Much better	124 (15.5%)	52 (22.0%)
2. Somewhat better	204 (25.5%)	47 (19.9%)
3. About the same	237 (29.6%)	57 (24.2%)
4. Somewhat worse	97 (12.1%)	34 (14.4%)
5. Much worse	138 (17.3%)	46 (19.5%)
Mean (SD)	2.90 (1.30)	2.89 (1.41)

#### 3.5.1 Duration

Of the recipients reporting improved mood, 25.5% reported that the improvement lasted less than a month, and 44.0% reported less than 3 months. The corresponding findings for reports by family/friends were 18.2% and 32.3%.

### 3.6 During the Treatment, What Effect Did ECT Have on How Suicidal You Felt?

Table 8 shows that 32.6% of recipients felt ECT reduced how suicidal they had felt; 48.6% reported ECT made no difference and 18.7% reported that ECT increased suicidality. When analysing only the 524 who reported ‘suicidal thoughts or feelings in the month before your last ECT series,’ the percentage reporting ‘less suicidal’ rose slightly, to 40.1%, but ‘more suicidal’ remained about the same (17.9%).

**TABLE 8:** During the treatment, what effect did ECT have on how suicidal X felt?

	Recipients (*n* = 763)	Family/Friends (*n* = 214)
Less suicidal	249 (32.6%)	75 (35.0%)
No difference	371 (48.6%)	98 (45.8%)
More suicidal	143 (18.7%)	41 (19.2%)

The reports of family/friends were very similar. About two thirds of both recipients (67.3%) and family/friends (65.0%) did not think ECT reduced suicidality (Table 8).

#### 3.6.1 Duration

Of the minority of recipients who reported reduced suicidality, 17.0% reported that the improvement lasted less than a month, and 33.2% reported less than 3 months. The corresponding findings for reports by family/friends were: 16.0% and 25.3%.

### 3.7 Electrode Placement

Table 9 shows that all five measures of effectiveness were, to varying degrees, positively related to unilateral ECT, compared to bilateral. For example, unilateral electrode placement was significantly related to recipients experiencing ECT as ‘helpful, overal’ (*X* ^2^ = 12.7, df = 3, *p* = 0.005). 40.6% of those respondents whose last course was unilateral ECT experienced ECT as ‘helpful, overall’, to some extent, compared to 29.5% for recipients of bilateral ECT.

**TABLE 9:** Strength of positive relationship (*χ* ^2^) with unilateral electrode placement, compared to bilateral placment.

	Recipients^a^	Recipients who had only one course^b^	Family/Friends^c^
Problem/symptoms	4.8	7.8	37.0**
Mood	9.7*	7.5	21.5*
Helpful, overall	12.7**	8.2*	21.9**
Quality of life	13.8*	18.6**	39.4**
Suicidality	10.4**	14.4***	12.1

^a^ *n* = between 489 and 514.

^b^ *n* = between 159 and 162.

^c^ *n* = between 209 and 241.

*< 0.05, **< 0.01, ***< 0.001.

When recipients who only received one course of ECT were analysed separately, unilateral ECT was strongly related to better Quality of Life and less Suicidality. For example, 21.3% of people whose only course involved bilateral ECT reported increased suicidality, compared to 10.8% for unilateral. (The numbers for family/friends were too small to warrant analysis).

## 4 Discussion

Our study confirms previous studies' findings that some ECT recipients find ECT to be effective, and shows that this is also the case for the reports of some family members and friends. The self‐reports of several hundred ECT recipients, however, indicate that on all five effectiveness measures the majority received either no benefit or a worsening of the variable measured. The percentages reporting some benefit were: the specific problem for which ECT was given—44.5%, mood—41.0%, helpful in general—41.5%, quality of life—29.1% and suicidality 32.6%. Furthermore, about one in six of those who reported reduced suicidality and one in four of those reporting improved mood reported relapse of the gain within a month.

An intervention that is reported to be effective by 29%–45% of patients cannot be dismissed despite its being judged ineffective for between 71% and 55%. However, for four of the five variables, respondents were, unusually, given the option to report that the variable had been made worse by ECT. The results were: problem made worse—36.6%, worse mood—29.4%, lower quality of life—62.3% and increased suicidality—18.9%. If one were to deduct the negative outcomes from the positives, the net results would be: problem +7.9%, mood +11.6%, quality of life −33.2%, suicidality +13.7%.

A finding that one in three report reduced suicidality needs to be balanced against the fact that one in five reported feeling *more* suicidal. (The latter may be an underestimate if some people who felt more suicidal did kill themselves as a result.)

It is particularly striking that the Quality of Life of 62.3% of recipients was made worse by ECT. Nearly half (49.1%) reported ‘much worse’ (21.6%) or ‘very much worse’ (27.5%). This finding was consistent with the reports from family/friends (see Table 6). Quality of life produced a net negative of 33.2%. A partial explanation of this concerning outcome is that Quality of Live encompasses the adverse effects of ECT alongside benefits.

Patient‐reported outcome measures (PROMs), including quality of life, are increasingly used in everyday clinical practice (Carfora et al. 2022; National Collaborating Centre for Mental Health 2023) and tend to involve measuring a broader range of issues of importance to patients, rather than just the traditional focus of many clinicians on symptom reduction.

It should also be noted that a very common response, for all five variables, was ‘minimal’ or no change. For example, the modal response concerning mood change was ‘About the same’ for both recipients (29.6%) and family/friends (24.2%). Nearly half of recipients (48.6%) and family/friends (45.8%) reported that ECT made no difference to suicidality. Similarly, 44.3% of recipients and 37.5% of family/friends reported that ECT had no or ‘minimal’ impact on the problem for which it was administered.

### 4.1 Family/Friends

The results from ECT recipients and family/friends were remarkably similar for all variables. This suggests that the recipients' responses should not be dismissed as unreliable.

### 4.2 Electrode Placement

Unilateral ECT, in which the two electrodes are placed on the same side of the brain, was introduced to reduce memory loss. However, it was discovered that it also tends to be less effective than bilateral in terms of reducing depression. The current survey found, among ECT recipients who had had only one course of ECT, that electrode placement was not related to improving mood or to the problem for which the ECT was prescribed. However unilateral ECT was significantly more effective in terms of quality of life, or, more accurately, it caused less deterioration therein. Presumably this is because quality of life is affected by the memory loss and other adverse effects. Similarly, unilateral performed better than bilateral in terms of reducing suicidality, which also might be increased by loss of memory and overall reduced life quality. An anecdotal example of this possible explanation is that shortly after ECT, Ernest Hemingway asked, just before he died by suicide: ‘What is the sense of ruining my head and erasing my memory, which is my capital, and putting me out of business? It was a brilliant cure, but we lost the patient’ (Hotchner 1967).

Despite bilateral ECT causing more memory loss a survey of 70 countries found that the ‘worldwide preferred electrode placement was bilateral’ (Leiknes et al. 2012). This was confirmed by 78% of ECT recipients reporting, in the current survey that their last course had been bilateral ECT.

### 4.3 Limitations

This survey relies on self‐reports, not validated outcome measures. The self‐reports relied on memory, which, in this area, may be more unreliable than usual. However, the fact that results from family/friends were remarkably similar to those from ECT recipients represents, perhaps, a modicum of (inter‐rater) reliability for the patients' reports.

The questionnaire was not subjected to construct validation, psychometric evaluation or pilot testing (beyond seeking feedback on a draft from Mind—see Section 2.1). Some of the outcome constructs, such as ‘helpful’ and ‘quality of life’ can be interpreted differently by individuals.

We did not recruit adequately from beyond English‐speaking countries in North America, Europe and Australasia. The survey was written in English without translations.

#### 4.3.1 Sample Biases

Sample bias towards people for whom ECT had a *positive* outcome was potentially present in four forms:

- Those for whom ECT had failed to alleviate the severe depression (for which it is typically prescribed) might be uninterested in, or unable to complete, a survey.
- Those whose suicidality was not alleviated by ECT, and who ended their lives themselves, did not participate.
- Patients who died during or soon after treatment due to cerebral or cardiovascular events (Duma et al. 2017; Read 2024) did not participate.
- Some of those in whom ECT caused severe cognitive damage would have been unable to participate.

Sample bias towards those for whom ECT had a *negative* outcome may have occurred from the dissemination of the survey on social media by the researchers, some of whom have critiqued ECT in research papers and online. To minimise this, social media posts frequently included phrases like ‘Positive, mixed and neutral experiences all equally valued’.

The same two types of bias had the potential to influence online surveys of other psychiatric treatments using the same methodology as the current study (Cartwright et al. 2016; Larsen‐Barr et al. 2018; Moncrieff et al. 2024; Read et al. 2014, 2016, 2017; Read, Morrison, et al. 2023; Read and Sacia 2020; Read and Williams 2019). All of these studies, however, elicited a wide range of views and experiences, as did the current survey. When asked ‘what effect did ECT have on your mood’, 41% of recipients endorsed ‘better’ or ‘much better’, 30% selected ‘no difference’ and 29% chose ‘worse’ or ‘much worse’.

A study of internet‐based surveys in the English NHS found that ‘patients’ website ratings of hospitals and more conventional measures of patient experience from large random surveys are significantly correlated (Greaves et al. 2012). It concluded ‘Our findings add to the increasingly persuasive literature promoting the notion that one needs to view safety, quality and service delivery through a number of lenses to get an accurate picture’.

## 5 Conclusion

Any cost–benefit analysis of a treatment considers not only the percentage of people who improve or deteriorate on effectiveness measures, but also the known adverse effects. A large, prospective study concluded that adverse cognitive effects can persist for an extended period, and that they characterize routine treatment with ECT in community settings (Sackeim et al. 2007). The American Psychiatric Association (APA) (2025) recently acknowledged that ‘some individuals may report having memory problems that remain for months or years, or even permanently.’ A prominent Psychiatry textbook states that ‘All patients should be informed that permanent memory loss may occur’ (Black and Andreasen 2011). An international manufacturer of ECT machines in the US has added a warning that their product can cause ‘permanent memory loss or permanent brain damage’ (Schwartzkopff 2018; Somatics 2018, 4). A recent joint report by the World Health Organisation and the United Nations (2023) argued that ‘People being offered ECT should also be made aware of all its risks and potential short‐ and long‐term harmful effects, such as memory loss and brain damage’. (p. 58).

Our own survey used four measures of memory problems, producing rates from 61% to 84%. Furthermore, 65% of those experiencing anterograde amnesia, and 81% of those reporting retrograde amnesia, reported that the deficit lasted at least 3 years. Family/friends also reported very high, but slightly lower, percentages of memory loss (Read et al., 2025). Seventeen other adverse effects were reported by more than 50% of ECT recipients, including Fatigue, Emotional blunting and Relationship problems.

Some of our results may be uncomfortable or confronting for some, but this is information that mental health nurses need to be aware of in order to support and inform people considering ECT. All mental health professionals, including nurses, have a shared responsibility to implement the ethical principle of informed consent. Recent audits in the UK (Harrop et al. 2021; Read, Morrison, et al. 2023) and Australia (Wand et al. 2024) have found that ECT information leaflets for patients and families routinely minimise adverse effects and exaggerate benefits. The current survey found that most (60%) of the ECT recipients reported that they had not been given ‘adequate information’. (Read, Harrop, Morrison, et al. 2025). Nurses will benefit from informing themselves about the research on the safety and efficacy of ECT in order to be in a position to play their part in the informed consent process.

In the meantime, we call for a suspension of ECT in clinical settings, pending the following (all of which are urgently needed with or without a suspension):

- Several independent, large placebo‐controlled studies to determine whether ECT has any effectiveness relative to placebo, at the end of treatment and at various follow‐up times, against which the adverse effects can be weighed in cost–benefit analyses.
- Several large studies monitoring cognitive and other adverse effects with appropriate measures, tracking outcomes for three years, to be conducted by government or University bodies.
- The development of an evidence‐based information document for patients and families which includes the high risk of permanent, severe memory dysfunctionand other neurological, psychological and social consequences.
- A commitment to routinely monitoring for memory and other cognitive problems during and after courses of ECT, using appropriately sensitive, comprehensive tests.
- The development of standard practices that include dosing consensus protocols.
- The offer of cognitive assessment and, where necessary, rehabilitation, for past, present and future ECT recipients.

## Author Contributions

All authors listed meet the authorship criteria according to the latest guidelines of the International Committee of Medical Journal Editors, and are in agreement with the manuscript.

## Disclosure

John Read has been a paid expert witness in several ECT legal cases in the USA and Canada.

## Conflicts of Interest

The authors declare no conflicts of interest.

## Data Availability

The data that support the findings of this study are available on request from the corresponding author.
